# Ozone detoxification of steam-pretreated Norway spruce

**DOI:** 10.1186/s13068-015-0388-7

**Published:** 2015-11-26

**Authors:** Adnan Cavka, Anna Wallenius, Björn Alriksson, Nils-Olof Nilvebrant, Leif J. Jönsson

**Affiliations:** Department of Chemistry, Umeå University, Linnaeus v. 10, 901 87 Umeå, Sweden; SP Processum AB, 891 22 Örnsköldsvik, Sweden; Borregaard, 1701 Sarpsborg, Norway

**Keywords:** Detoxification, Lignocellulose, Ozone, *Saccharomyces cerevisiae*, Fermentability

## Abstract

**Background:**

Pretreatment of lignocellulose for biochemical conversion commonly results in formation of by-products that inhibit microorganisms and cellulolytic enzymes. To make bioconversion processes more efficient, inhibition problems can be alleviated through conditioning. Ozone is currently commercially employed in pulp and paper production for bleaching, as it offers the desirable capability to disrupt unsaturated bonds in lignin through an ionic reaction known as ozonolysis. Ozonolysis is more selective towards lignin than cellulose, for instance, when compared to other oxidative treatment methods, such as Fenton’s reagent. Ozone may thus have desirable properties for conditioning of pretreated lignocellulose without concomitant degradation of cellulose or sugars. Ozone treatment of SO_2_-impregnated steam-pretreated Norway spruce was explored as a potential approach to decrease inhibition of yeast and cellulolytic enzymes. This novel approach was furthermore compared to some of the most effective methods for conditioning of pretreated lignocellulose, i.e., treatment with alkali and sodium dithionite.

**Results:**

Low dosages of ozone decreased the total contents of phenolics to about half of the initial value and improved the fermentability. Increasing ozone dosages led to almost proportional increase in the contents of total acids, including formic acid, which ultimately led to poor fermentability at higher ozone dosages. The decrease of the contents of furfural and 5-hydroxymethylfurfural was inversely proportional (*R*^2^ > 0.99) to the duration of the ozone treatment, but exhibited no connection with the fermentability. Ozone detoxification was compared with other detoxification methods and was superior to treatment with Fenton’s reagent, which exhibited no positive effect on fermentability. However, ozone detoxification was less efficient than treatment with alkali or sodium dithionite. High ozone dosages decreased the inhibition of cellulolytic enzymes as the glucose yield was improved with 13 % compared to that of an untreated control.

**Conclusions:**

Low dosages of ozone were beneficial for the fermentation of steam-pretreated Norway spruce, while high dosages decreased the inhibition of cellulolytic enzymes by soluble components in the pretreatment liquid. While clearly of interest for conditioning of lignocellulosic hydrolysates, future challenges include finding conditions that provide beneficial effects both with regard to enzymatic saccharification and microbial fermentation.

## Background

Biochemical conversion of lignocellulosic feedstocks typically includes a thermochemical pretreatment step which primarily targets the hemicellulose, an enzymatic saccharification of the cellulose, and a microbial fermentation step in which sugars are converted to advanced biofuels, green chemicals, or other desirable products [1]. With powerful pretreatment methods, such as steam explosion with acid catalysts, also recalcitrant lignocellulosic feedstocks including softwood can be made susceptible to cellulolytic enzymes [2–4]. Harsh pretreatment under acidic conditions leads to the formation of by-products that inhibit enzymic and microbial biocatalysts [5, 6]. Microbial inhibitors commonly studied include phenolic compounds, aliphatic carboxylic acids (such as acetic acid, formic acid, and levulinic acid), and furan aldehydes [such as furfural and 5-hydroxymethylfurfural (HMF)].

The detrimental effects of inhibitory compounds on microbial biocatalysts can be alleviated through conditioning of lignocellulosic hydrolysates by chemical detoxification with, for example, alkali or reducing agents [6]. Efficient detoxification, such as alkaline treatment and treatment with sodium dithionite, renders strongly inhibitory hydrolysates as fermentable as reference fermentations with the same amounts of sugar but no inhibitors. It is, however, important that the detoxification is selective and targets inhibitors rather than fermentable sugars. For example, treatment with alkali has to be optimized carefully to achieve an efficient detoxifying effect without any major loss of fermentable sugars [6, 7]. Some reducing agents, such as sodium dithionite, can also alleviate inhibition of cellulolytic enzymes, while others, such as sodium borohydride, do not have that effect [8].

Previous investigations indicate that phenolic compounds inhibit both microorganisms, such as yeast [9, 10] and cellulolytic enzymes [11, 12]. Phenol-oxidizing enzymes, such as laccase and peroxidase, can be used to decrease the inhibitory effects of phenols [9, 10]. In this investigation, we have assessed the possibility of using treatment with ozone to alleviate inhibitory effects of phenolic and other aromatic compounds. Ozone treatment under acidic conditions has been found to be useful for bleaching of pulp in the pulp and paper industry [13]. Studies regarding the use of ozone for pulping have shown that a potential advantage with ozone treatment is selectivity, as the rate constants for ozone oxidation of aromatic compounds are typically several orders of magnitude higher than the rate constants for ozone oxidation of simple sugars [13]. Thus, we hypothesized that ozone treatment could be used to target aromatic substances in hydrolysates without causing significant degradation of fermentable sugars. The effects of treatments with different ozone dosages on a hydrolysate of pretreated spruce wood were investigated, and a comparison with other detoxification methods was made to put ozone treatment in a larger context within the bioconversion area.

## Results and discussion

The effects of ozone treatment on fermentation inhibitors formed during pretreatment of lignocellulose were investigated using pretreatment liquid of Norway spruce. While it is necessary that detoxification improves biocatalytic performance, it is important that fermentable sugars are not degraded as a result of the treatment, which would lead to decreased product yields. Alkali treatment may result in sugar degradation, which, however, can be minimized through optimization [6, 7, 14]. Treatment with reducing agents such as sodium dithionite does not result in sugar degradation [15]. A recent report on detoxification with Fenton’s reagent did not address sugar degradation [16]. Preferably, detoxifying agents should be selective for inhibitors allowing for minimal dosage of the detoxifying agent as well as minimal sugar degradation.

### Effects of ozone on fermentable sugars

Monomeric sugars in the pretreatment liquid were primarily derived from hemicelluloses, which in Norway spruce mainly consist of galactoglucomannans [17]. The pretreatment liquid contained (in g/L) glucose, 29; mannose, 29; xylose, 15; galactose, 6; and arabinose, 5. Analysis of monosaccharides was also performed after ozone was applied to the pretreatment liquid in different dosages ranging from 0.5 to 3.5 g. The results showed that no sugar degradation had occurred during ozone treatment of the pretreatment liquid. The monosaccharide concentrations in ozone-treated samples differed less than 5 %, which is below the estimated standard deviation of the analytical method used.

Detoxifying agents with high selectivity are needed to assure reactions with inhibitors without affecting fermentable sugars. The reaction rates of ozone treatment of different organic substrates have been found to differ by several orders of magnitude when compounds representing polysaccharides and lignin are compared, for example, glucose and phenol [13, 18]. Ozone has also been shown to be between 10^5^ and 10^6^ times more reactive than hydroxyl radicals towards lignin [19]. The hydroxyl and hydroperoxy radicals formed by ozone decomposition and by Fenton’s reagent have on the contrary been shown to be far more reactive and much less selective than ozone [20, 21]. The formation of hydroxyl radicals from ozone is, however, strongly pH dependent as the formation of hydroxyl radicals from ozone starts to become extensive at pH ≥ 3 [22, 23]. All treatments in this study were performed at an initial pH of 2.5, which could tentatively explain the absence of carbohydrate degradation even at the highest ozone load that was applied.

### Effects of ozone on inhibitory compounds

The effects of ozone treatment on inhibitory by-products that were formed during pretreatment were also investigated. Four levels of ozone addition, i.e., 0.5, 1.5, 2.5, and 3.5 g, were selected for the analysis in addition to the untreated pretreatment liquid. Inhibitory pretreatment by-products that were analyzed included the total amount of phenolic compounds, prevalent small aliphatic carboxylic acids (acetic acid, formic acid, and levulinic acid), the total concentrations of acids, and the furan aldehydes furfural and HMF (Table 1).

**Table 1:** Effects of ozone treatment on inhibitory compounds in spruce pretreatment liquid

Compounds	Untreated	0.5 g	1.5 g	2.5 g	3.5 g
Phenolics	32.2	21.7	17.1	17.1	16.4
Acetic acid	111	112	111	112	113
Formic acid	17.9	21.8	29.1	32.1	37.5
Levulinic acid	9.3	9.3	9.2	9.3	9.4
Total acids	222	251	285	305	326
Furfural	24.5	21.6	16.3	10.3	6.5
HMF	16.4	14.8	11.9	8.4	5.6

The table shows the concentrations of common inhibitors and groups of inhibitors in spruce hydrolysate (mM) before and after treatment with different ozone dosages

**Fig. 1:**
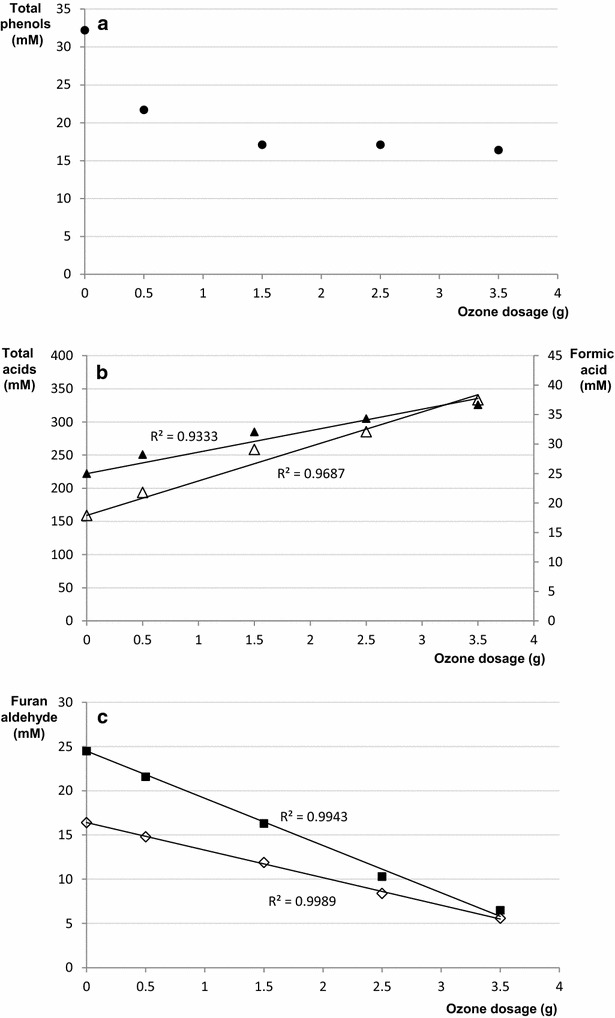
Effects of increasing ozone dosages on **a** the total phenolic contents determined by the Folin–Ciocalteu assay (*closed circle*), **b** formic acid (*open triangle*) and the total acid contents (*closed triangle*), and **c** furfural (*closed square*) and HMF (*open diamond*)

The Folin–Ciocalteu assay indicated that the total concentration of phenols in the pretreatment liquid decreased significantly even at lower ozone dosages (Table 1; Fig. 1a). The total phenolic content was determined to be 32.2 mM in the pretreatment liquid and addition of increasing amounts of ozone decreased that concentration until it was about half of the initial (Table 1; Fig. 1a). According to the results obtained with the Folin–Ciocalteu assay, higher ozone dosages than 1.5 g did not result in any further decrease of the total phenolic content (Table 1; Fig. 1a).

Studies of the mechanism behind the reactions of ozone with lignin suggest that the primary mode of action of ozone is degradation of unsaturated structures through the classical Craigee reaction [24, 25]. The reaction, which involves the addition of ozone across an unsaturated structure followed by a subsequent fragmentation of the intermediate into carbonyl-containing products, may result in the formation of hydroxyl radicals [13, 26]. Hydroxyl radicals are not selective, as ozone, and would most probably attack other compounds in the pretreatment liquid than lignin-derived aromatic compounds, for instance, carbohydrates. Step-wise ozone degradation of low molecular weight model compounds suggests that the degradation of lignin results in products with muconic acid structures as well as in formation of methanol, formic acid, and carbon dioxide [13, 17]. Investigations in this area cover reactions with individual mononuclear phenolic compounds [13, 25, 26].

The analysis of small aliphatic acids in the pretreatment liquid and the ozone-treated samples showed that the concentrations of acetic acid and levulinic acid were unaffected by the addition of ozone to the pretreatment liquid (Table 1). The concentrations of acetic acid were around 110 mM in all of the analyzed samples, while the concentrations of levulinic acid were just above 9 mM. In contrast with acetic acid and levulinic acid, the concentration of formic acid increased with increasing ozone dosage (Table 1; Fig. 1b). The highest ozone dosage resulted in a formic acid concentration that was over 100 % higher than in the untreated sample. With an *R*^2^ value of about 0.97, the increase was almost proportional to the ozone dosage (Fig. 1b). These results are consistent with previous studies indicating that formation of formic acid, methanol, and carbon dioxide occurs when phenolic compounds decompose as a result of ozonolysis [13, 17].

The strong increase of the concentration of formic acid and the probable formation of muconic acids during ozone treatment motivated the determination of the total acid content through titration with sodium hydroxide. The results showed that the total acid content in the pretreatment liquid was around 220 mM (Table 1), which increased with about 50 % at the highest ozone dosage. The rate of increase of the total acid content was slightly higher at smaller than at larger ozone additions resulting in an *R*^2^ value of about 0.93 (Fig. 1b). The results further show that the three small aliphatic carboxylic acids that were quantified separately (acetic acid, formic acid, levulinic acid) constitute roughly 60 % of the total acid content of the pretreatment liquid.

The results of the HPLC-UV-DAD analysis further showed that the concentrations of HMF and furfural steadily decreased with increasing additions of ozone to the pretreatment liquid (Table 1; Fig. 1c). The decrease was inversely proportional to the ozone dosage (*R*^2^ > 0.99, Fig. 1c). However, the contents of furan aldehydes may be affected not only by chemical reactions with ozone, but also by evaporation. Larsson et al. [10] studied the effects of evaporation on a hydrolysate and found that an evaporation of 10 % of the volume led to a furfural decrease of 37 %, while the concentration of HMF was not affected. When the evaporation was increased to 90 % of the volume, there was no furfural left, while the decrease of the content of HMF was only 4 % [10]. Thus, furfural is much more susceptible to evaporation than HMF. In the ozone experiment, the rate of decrease of furfural was higher than the rate of decrease of HMF (Fig. 1c), which could tentatively be attributed to evaporation. However, the fact that there was a steady decrease in the contents of HMF (Table 1; Fig. 1c) indicates that reactions between furan aldehydes and ozone were also taking place. Considering the unsaturated structure of the furan aldehydes, this would be consistent with the previously discussed Craigee reaction. At the lowest ozone dosage (0.5 g), the combined decrease of furfural and HMF amounted to only 4.5 mM compared with 10.5 mM for the total phenolic content despite the fact that the initial combined concentration of the furan aldehydes (40.9 mM) was higher than the initial total phenol concentration (32.2 mM) (Table 1). The total phenolic content is not much affected by evaporation [10]. Taken together, these results indicate that the furan aldehydes were less susceptible than the phenols to lower ozone dosages.

In summary, ozone treatment decreased the concentrations of phenolic compounds and furan aldehydes while increasing the concentrations of certain carboxylic acids, such as formic acid. Phenolic compounds were more strongly affected than furan aldehydes at lower ozone dosages. Further increases of the ozone dosage led to further decrease of the concentrations of furan aldehydes, but not of the remaining phenolic content, as judged by the Folin–Ciocalteu assay.

### Effects of ozone on fermentation

**Fig. 2:**
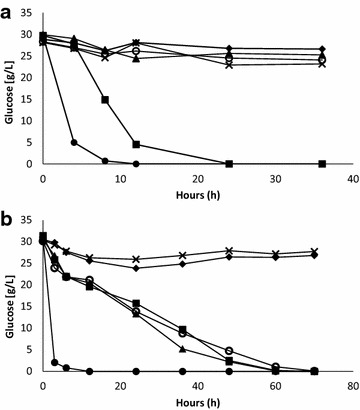
Glucose consumption by *S. cerevisiae* during the fermentation of pretreatment liquid produced from Norway spruce. *Markers* in **a** sugar reference (*closed circle*), untreated (*closed diamond*), 0.5 g ozone (*closed square*), 1.5 g ozone (*cross symbol*), 2.5 g ozone (*closed triangle*), 3.5 g ozone (*open circle*). *Markers* in **b** sugar reference (*closed circle*), untreated (*closed diamond*), 0.25 g ozone (*open circle*), 0.5 g ozone (*closed square*), 0.75 g ozone (*closed triangle*), and 1.5 g ozone (*cross symbol*)

The observed effects of ozone addition on inhibitory pretreatment by-products merited further investigation into the potential effects on glucose consumption and ethanol formation by the yeast *Saccharomyces cerevisiae*. Fermentation experiments conducted with ozone additions of 0.5, 1.5, 2.5, and 3.5 g showed that only 0.5 g of ozone resulted in glucose consumption and ethanol production (Fig. 2a). Increased dosages of ozone did not result in much larger decreases of the content of phenolic compounds (Table 1), which suggests that the rapid initial decrease in the contents of phenolic compound is linked to the improved fermentability. Although increased ozone dosages led to a strong decrease of the contents of furan aldehydes (Fig. 1c), there was no beneficial effect on the fermentability (Fig. 2a). The fact that the concentration of formic acid and the total contents of acids rose to high levels when the dosage of ozone was 1.5 g or higher (Table 1) can explain why the fermentability was poorer than when the ozone dosage was 0.5 g. The results support the advocated importance of phenolic compounds and small aliphatic carboxylic acids for the inhibition of *S. cerevisiae* in pretreated lignocellulosic feedstocks [6].

A second set of experiments was performed with ozone additions of 0.25, 0.5, 0.75, and 1.5 g to further investigate potential positive effects of moderate to low ozone concentrations on the fermentability. The glucose consumption in the second set of fermentation experiments is shown in Fig. 2b. Ozone dosages of 0.25, 0.5, or 0.75 g resulted in similar fermentability. In contrast, 1.5 g did not result in any improvement indicating a very sharp threshold between 0.75 and 1.5 g. The results show that moderate to low additions of ozone have a positive effect on the glucose consumption by *S. cerevisiae* and that the positive effects were achieved already at the lowest ozone dosage tested, i.e., 0.25 g. Fermentations were performed at a starting pH of 6.0 but without pH adjustment during the fermentation process. Assuming that undissociated acids cause more inhibition problems, there is a possibility that control of pH during the fermentation process could alleviate problems with formation of inhibitory carboxylic acids formed by ozone treatment by keeping the acids in a deprotonated state.

### Comparison of detoxification methods

**Fig. 3:**
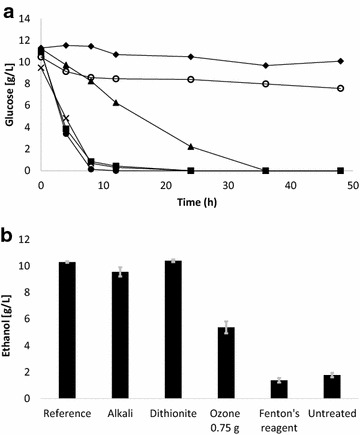
Comparison of detoxification methods: **a** glucose consumption, and **b** ethanol production after 24 h of fermentation. The *markers* indicate: untreated (*closed diamond*), alkali detoxification (*cross symbol*), sodium dithionite (*closed square*), ozone (0.75 g) (*closed triangle*), Fenton’s reagent (*open circle*), and reference fermentation (*closed circle*). *Gray error* *bars* show standard deviations

Three additional detoxification methods were chosen and included in a fermentation experiment designed to benchmark the improvement in glucose consumption and ethanol productivity by *S. cerevisiae* after ozone treatment of spruce wood pretreatment liquid. Treatments with alkali, such as ammonium hydroxide, and with reducing agents, such as dithionite, are powerful detoxification methods that have previously been shown to result in a fermentability that is similar to that of a reference fermentation with equal concentrations of fermentable sugar but without any inhibitors [6, 8, 14, 15]. Fenton’s reagent was included as there is a claim in the literature that it would have a strongly positive effect on the fermentability of spruce wood hydrolysates [16] and since it is, as ozone, based on reactive oxygen species. The results of the comparison are shown in Fig. 3 and Table 2. As expected [6, 8, 14, 15], detoxification with sodium dithionite and ammonium hydroxide resulted in a glucose consumption rate that strongly resembled the rate observed for the reference fermentation that did not contain any inhibitory compounds. Ethanol titers (Fig. 3b), ethanol yields, balanced ethanol yields, and ethanol productivities (Table 2) were also very similar for the reference and the treatments with alkali and dithionite. Persson et al. [27] showed that the effects of alkali treatment are due to chemical conversion of inhibitors rather than to precipitation effects. This was later confirmed in experiments with alkali treatment through addition of sodium hydroxide, which did not result in any precipitate and yet strongly improved the fermentability [14]. Dithionite and other sulfur oxyanions, such as sulfite, effect detoxification through sulfonation of aromatic compounds which makes them less reactive and strongly hydrophilic [28].

**Table 2:** Comparison of detoxification methods for improved fermentability of spruce pretreatment liquid

	Fermentable hexoses (glucose and mannose) at start (g/L)	Fermentable hexoses (glucose and mannose) after 24 h (g/L)	Consumed sugar (glucose and mannose) (g/L)	Ethanol yield^a^	Balanced ethanol yield^b^	Ethanol productivity^c^
Reference	20.3	<0.01	20.3	0.51	0.51	0.43
Untreated	20.6	16.5	4.1	0.43	0.09	0.07
Alkali	19.0	<0.01	19.0	0.50	0.50	0.40
Dithionite	21.0	<0.01	21.0	0.49	0.49	0.43
Ozone 0.75 g	20.3	8.4	11.9	0.45	0.26	0.22
Fenton’s reagent	20.8	17.0	3.8	0.37	0.07	0.06

The table compares the concentrations of fermentable hexose sugars at the start of the experiment and after 24 h of fermentation after detoxification using different methods. The relative standard deviation of the method that was used for monosaccharide and ethanol analysis was estimated to <10 %

^a^g EtOH/g consumed glucose and mannose

^b^g EtOH/g glucose and mannose prior to fermentation

^c^g EtOH × L^−1^ × h^−1^

In contrast with previously reported results [16] and despite using the reagent concentrations reported to be optimal for similarly pretreated Norway spruce, fermentations with Fenton’s reagent did not result in any noteworthy improvements of glucose consumption or ethanol production compared to the untreated control fermentation (Table 2; Fig. 3). Both sets of fermentations resulted in low glucose consumption, i.e., around 4 g/L, and ethanol titers of less than 2 g/L. For the untreated control and the treatment with Fenton’s reagent, the balanced ethanol yields and the ethanol productivities were similar, and far below those achieved in other fermentations (Table 2). Even the ethanol yields on consumed sugar were the lowest observed in the experimental series (Table 2).

The poor performance of the Fenton’s reagent is not unexpected considering the strong reactivity and poor selectivity of hydroxyl radicals. Formation of hydroxyl radicals in pulp bleaching with hydrogen peroxide is decreased through addition of chelating agents, such as EDTA (ethylenediaminetetraacetic acid) and DTPA (diethylene triamine pentaacetic acid), which chelate Mn(II) and Fe(II), ions that could otherwise be involved in Fenton-type reactions [29]. Decreasing Fenton-type reactions is advantageous, as the poor selectivity of hydroxyl radicals would lead to reactions with carbohydrates as well as lignin, and therefore to reduced pulp yields [29]. High concentrations of fermentable sugar would also be attacked by hydroxyl radicals leading to a loss of fermentable sugars. Ozone, on the other hand, is useful in pulp bleaching due to that it is more selective, which makes it possible to use conditions where lignin structures are oxidized with minimal effects on the cellulose fibers. With regard to lignocellulosic hydrolysates, one would expect similar effects, where Fenton’s reagent is involved in reactions with various organic matter in the pretreatment liquid, including sugars, while, as shown in this work, ozone can selectively degrade substances such as phenolics and furans. It is also possible that the Fenton’s reaction was not efficient in the complex lignocellulosic medium. Due to its poor performance, the effects of treatment with Fenton’s reagent were not further investigated.

The glucose consumption and ethanol titers in fermentations of ozone-treated medium clearly exceeded those of untreated hydrolysate and the treatment with Fenton’s reagent, but were not comparable with fermentation of hydrolysates treated with sodium dithionite or ammonium hydroxide (Fig. 3). The ethanol yield on consumed sugar, 0.45 g/g, was slightly lower than for the reference fermentation and for hydrolysates treated with alkali or dithionite (Table 2). As high concentrations of carboxylic acids may result in a high ethanol yield due to increased ethanol formation at the expense of biomass formation (reviewed in [6]), this was somewhat unexpected, but it can probably be attributed to that the ethanol yields of the reference, the alkali-treated hydrolysate, and the dithionite-treated hydrolysate were at or very near the theoretical maximum at 0.51 g/g. The balanced ethanol yield and the ethanol productivity for ozone-treated hydrolysates were around half of those reached with the reference and alkali- or dithionite-treated hydrolysate.

While high concentrations of carboxylic acids can be problematic to the fermenting microorganism, they may not be as toxic on a molar basis as the phenolic compounds that can serve as a source of muconic acids and formic acid formed through ozone treatment. This may help explain the improvement compared to the untreated control at lower ozone dosages (up to 0.75 g), as well as the decrease in the improvement of fermentability when higher ozone dosages (1.5 g or higher) were applied. Similarly, too harsh conditions during alkaline detoxification lead to an increase of phenolic compounds, formic acid, and acetic acid, and, in turn, sub-optimal detoxification effects [7].

### Effects of ozone on saccharification

**Fig. 4:**
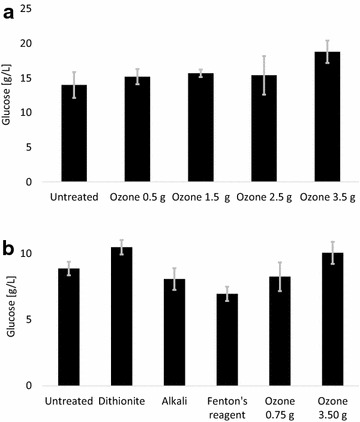
Enzymatic hydrolysis of Avicel in the presence of pretreatment liquid: **a** different ozone dosages, and **b** comparison of different methods. *Gray error bars* show standard deviations

Reducing agents, such as dithionite, can have a positive effect on enzymatic hydrolysis of cellulose, while other reducing agents, such as sodium borohydride, do not have that effect [8]. As different conditioning methods have different effects on enzymatic hydrolysis of cellulose, the potential effects of ozone treatment on enzyme inhibition by the pretreatment liquid were studied and compared with the effects of the other detoxification methods. In a first experimental series, different ozone dosages were compared (Fig. 4a). While pretreatment liquid treated with ozone dosages ranging from 0.5 to 2.5 g resulted in similar amounts of glucose as the untreated control, the highest ozone dosage (3.5 g) clearly gave more glucose (Fig. 4a). The second experimental series compared the effects of high (3.5 g) and low (0.75 g) dosages of ozone with the effects of the other treatments, i.e., sodium dithionite, alkali, and Fenton’s reagent (Fig. 4b). As expected, treatments with sodium dithionite or 3.5 g ozone exhibited a positive effect on enzymatic hydrolysis of cellulose, as the glucose yield was 18 % higher for sodium dithionite and 13 % higher for 3.5 g ozone compared to the untreated control. Alkali treatment with ammonium hydroxide resulted in a similar glucose yield as the untreated control (Fig. 4b). In contrast with a report in the literature that Fenton’s reagent would have a positive effect on enzymatic hydrolysis [16], Fenton’s reagent instead exhibited a slightly negative effect (Fig. 4b). The slight negative effect of Fenton’s reagent can possibly be attributed to residual hydrogen peroxide inhibiting the enzymes. With regard to the ozone treatment, the experiments did not reveal any ozone dosage that was beneficial for both yeast and cellulolytic enzymes, as a dosage of 3.5 g, which was beneficial for enzymatic hydrolysis (Fig. 4b), did not have any positive effect on the fermentation (Fig. 2a).

## Conclusions

Ozone treatment of a hemicellulose hydrolysate improved yeast fermentation already at low dosages, while higher dosages were required to improve enzymatic hydrolysis of cellulose. Ozone treatment decreased the furan aldehyde concentrations, but the results showed no correlation between furan aldehydes and fermentability. Treatment with ozone was superior to treatment with Fenton’s reagent, which exhibited no positive effect on fermentability. The potential of ozone in industrial biorefineries is, however, still doubtful, as an alternative method based on sulfur oxyanions has several advantages, including no separate process step, stronger detoxification, and positive effects on both microbial and enzymatic biocatalysts already at low concentrations.

## Methods

### Pretreatment of Norway spruce

The pretreatment liquid used in this study was obtained through vacuum filtration of pretreated wood chips of Norway spruce (*Picea abies*) using a procedure in which the solids were separated from the liquid phase using a Whatman grade 1 filter paper (Sigma-Aldrich, St. Louis, MO, USA). The raw materials were thermochemically pretreated by SEKAB E-Technology in the Swedish Biorefinery Demonstration Plant (Örnsköldsvik, Sweden). Spruce wood chips were pretreated in continuous mode in a 30-L reactor at a pressure of 20 bar (210 °C), loaded to approx. 50 % during operation. There was an addition of 1.2 kg SO_2_/h, which corresponds to approximately 1 % (w/w) SO_2_/spruce wood chips (dry weight). The residence time in the reactor was 7 min, and the resulting pH was 1.5. After pretreatment, the spruce slurry was cooled and stored at 4 °C until further use.

### Ozone treatment of spruce pretreatment liquid

The ozone treatments were performed with a Fischer Technology Ozone Generator 500 (i-Fischer Engineering GmbH, Waldbüttelbrunn, Germany) producing a continuous flow of ozone. The flow meter of the ozone generator was set to an oxygen gas throughput quantity of 150 L/min, which was kept constant with a flow controller. The applied levels of ozone were based on a preliminary study performed on acid-hydrolyzed spruce wood chips that had resulted in improved fermentability and ethanol yields after treatment with ozone (unpublished results). In the present study, six different treatment levels of ozone were applied: 0.25, 0.50, 0.75, 1.50, 2.50, and 3.50 g. The added amounts of ozone, which reflect the amounts of ozone led through the bottle rather than the amounts that reacted with the hydrolysate, were determined through iodometric titration after allowing the generator to warm up and a stable production of ozone was achieved. The ozone addition was controlled by calculating the time needed to feed the desired amount of ozone to the sample bottle. Sample volumes of 100 mL were placed in 250-mL gas wash bottles to allow for continuous ozone flow. Ten μL of anti-foam was added to each bottle to prevent the flasks from overflowing during treatment. The residual ozone that had not reacted with the sample was lead through a connecting gas wash bottle containing 40 g/L KI in Na_2_HPO_4_/KH_2_PO_4_ buffer solution. Iodometric titration with 0.1 M Na_2_S_2_O_3_ solution and a starch indicator was performed after acidification with 2 N H_2_SO_4_. The ozone-exposed samples were all transferred to glass bottles that were sealed with parafilm and stored at 4 °C until further use.

### Detoxification with alkali, sodium dithionite, and Fenton’s reagent

Three previously reported detoxification methods were included in the study in order to benchmark the efficiency of the ozone treatment on lignocellulosic hydrolysates. Alkali detoxification was performed by treating the pretreatment liquid with NH_4_OH (pH 9, 55 °C, 3 h), as these are optimal conditions for alkali detoxification [14]. Sodium dithionite detoxification was performed by adding sodium dithionite powder to a final concentration of 10 mM directly to the fermentation vessel 10 min prior to the fermentation, according to Alriksson et al. [15]. Treatment with Fenton’s reagent was performed using 2.5 mM FeSO_4_ and 150 mM H_2_O_2_ at a temperature of 60 °C for 2 h, as this has been reported to be optimal conditions for detoxification of pretreated Norway spruce [16]. Deionized water was added to compensate for dilutions associated with some of the detoxification methods.

### Fermentation

Fermentation experiments with common baker’s yeast *S. cerevisiae* (Jästbolaget AB, Rotebro, Sweden) were conducted to assess the possible detoxification effects of ozone treatment on pretreatment liquid from Norway spruce. The fermentations were performed in 25-mL glass flasks equipped with magnets for stirring and sealed with rubber plugs pierced with cannulas for release of carbon dioxide. Untreated prehydrolysate controls and treated prehydrolysates were diluted to 50 % prior to fermentation experiments. The pH was adjusted to 6.0 using 5 M NaOH. Each fermentation flask was filled with 4.75 mL of untreated pretreatment liquid, or of a sugar reference solution containing similar concentrations of glucose and mannose as the pretreatment liquid, or of pretreatment liquid treated with 0.25, 0.50, 0.75, 1.25, 2.50, or 3.50 g ozone. Each flask was supplemented with 0.10 mL of a nutrient solution (150 g/L yeast extract, 75 g/L (NH_4_)_2_HPO_4_, 3.75 g/L MgSO_4_·7 H_2_O, 238.2 g/L NaH_2_PO4·H_2_O), and 0.15 mL of yeast inoculum to a final concentration of 2 g/L (DW). The yeast inoculum was prepared in 750-mL cotton-plugged shake flasks with 300 mL YPD medium (2 % yeast extract, 1 % peptone, 2 % d-glucose). The flasks were inoculated and incubated with agitation at 30 °C for approximately 12 h. The cells were harvested in the late exponential growth phase by centrifugation (Allegra X-22R, Beckman Coulter, Brea, CA, USA) at 1500*g* for 5 min. The cells were re-suspended in an appropriate amount of sterile water to achieve an inoculum consisting of 2 g/L (cell dry weight) in all fermentation vessels. The flasks were incubated at 30 °C in a water bath with magnetic stirring (IKA-Werke, Staufen, Germany). Samples for measurement of sugars and ethanol were withdrawn during the fermentation. The glucose levels during fermentation experiments were estimated using a glucometer (Accu-Chek Active, Roche Diagnostics, Basel, Switzerland). Glucometer data were corrected using glucose values obtained with the HPAEC ICS-3000 system.

### Effects on enzymatic hydrolysis

The potential effects of conditioning of the pretreatment liquid on the performance of a cocktail of cellulolytic enzymes were investigated in two separate experimental series. The goal with the first experimental series was to compare different ozone dosages, and the series comprised pretreatment liquid treated with 0.5, 1.5, 2.5, and 3.5 g ozone as well as a control that was not treated with ozone. The aim of the second experimental series was to compare different conditioning methods and included pretreatment liquid treated with sodium dithionite, ammonium hydroxide, Fenton’s reagent, 0.75 g ozone, 3.5 g ozone, and an untreated control. The enzymatic reactions were performed in 2-mL microcentrifuge tubes, and the total weight of the reaction mixture in each tube was 1000 mg. The reaction mixture consisted of 925 mg of pretreatment liquid, 50 mg Avicel (Fluka Biochemika, Buchs Switzerland), and 25 mg of a 1:1 (w/w) mixture of the two liquid enzyme preparations Celluclast 1.5 L, with a stated activity of 700 endoglucanase units (EGU)/g, and Novozyme 188, with a stated activity of 250 cellobiase units (CBU)/g (Sigma-Aldrich). The enzyme loads in the analytical saccharification experiment were thus 175 EGU/g of solids (DW) and 62.5 CBU/g of solids (DW), respectively. In the first experimental series, the reaction mixtures were incubated at 45 °C and 180 rpm (Ecotron, Infors AG, Bottmingen, Switzerland) for 120 h. In the second experimental series, the temperature was increased to 50 °C, while the incubation time was decreased to 24 h.

### Chemical analyses

Glucose, xylose, mannose, arabinose, and galactose were analyzed using the high-performance anion-exchange chromatography (HPAEC) ICS-3000 system from Dionex (Sunnyvale, CA, USA) with an electrochemical detector. All samples were filtered through 0.20-μm syringe-driven filter units (Millex-GN, Millipore, Ireland) and diluted with Milli-Q water prior to analysis. Separation was performed using a CarboPac PA20 (3 × 150 mm) separation column equipped with a CarboPac PA20 (3 × 30 mm) guard column (Dionex). Elution was conducted at a flow rate of 0.4 mL/min with 2 mM solution of NaOH during 25 min, followed by regeneration at 5 min with 100 mM NaOH, and equilibration for 15 min with 2 mM NaOH (NaOH solution for IC, Sigma-Aldrich).

Analyses of ethanol, formic acid, acetic acid, and levulinic acid were performed using an Agilent Technologies 1200 series high-performance liquid chromatography system (HPLC) (Agilent Technologies, Santa Clara, CA, USA). The device was equipped with an autosampler, refractive index detector (RID), a binary pump, and degasser all from the Agilent 1200 series. The chromatographic separations were performed using a Bio-Rad Aminex HPX-87H column (Bio-Rad Laboratories, Hercules, CA, USA). Separation was achieved with an isocratic gradient of 0.01 N sulfuric acid at a flow rate of 0.6 mL/min, and the temperature of the column oven was set to 60 °C and the temperature of the detector cell of the RID was set to 55 °C [30]. An external calibration approach was applied for quantification.

5-(Hydroxymethyl)-furfural (HMF) and furfural were analyzed using the same HPLC system but with an Agilent Zorbax RRHT SB-C18 (3.0 mm × 50 mm, 1.8 μm) column and UV G1315D-detector (Agilent Technologies). Separation was performed with a gradient of 0.1 % (v/v) formic acid in Milli-Q water (A) and 0.1 % (v/v) formic acid in acetonitrile (B) and a flow rate of 0.5 mL/min. Gradients were set to percentage ratios of 97/3 (A/B) for 0–3 min and 90/10 (A/B) for 3–5 min. Both compounds were detected at a wavelength of 282 nm. The column oven was set to a temperature of 40 °C.

Total acid concentrations in the untreated and ozone-treated pretreatment liquids were estimated through titration with 200 mM NaOH (701 KF Titrino Titrator, Metrohm AG, Herisau, Switzerland).

The total phenolics were estimated in pretreatment liquid before and after treatment with 0.50, 1.50, 2.50, and 3.50 g ozone using the Folin–Ciocalteu assay. Vanillin was used as a reference standard. Briefly, 0.2 mL of each of the treated samples was mixed with 6 mL of Milli-Q water and 0.6 mL of Folin–Ciocalteu reagent (Sigma-Aldrich) and incubated at room temperature for 6 min. Two mL of 20 % (w/v) Na_2_CO_3_ was subsequently added under stirring. The samples were incubated for 2 h at room temperature, after which the samples were filtered (0.2 μm pore size) before the absorbance at 760 nm was measured using a spectrophotometer (UV-1800, Shimadzu, Kyoto, Japan).

## Abbreviations

DTPA: diethylene triamine pentaacetic acid
EDTA: ethylene diamine tetraacetic acid
HMF: 5-hydroxymethylfurfural
HPAEC: high-performance anion-exchange chromatography
HPLC-UV-DAD: high-performance liquid chromatography-ultra-violet-diode array detector
*R*^2^: coefficient of determination
RID: refractive index detector
*S. cerevisiae*: *Saccharomyces cerevisiae*

## References

[1] Lynd LR, Laser MS, Bransby D, Dale BE, Davison B, Hamilton R, Himmel M, Keller M, McMillan JD, Sheehan J, Wyman CE (2008). How biotech can transform biofuels. Nat Biotech.

[2] Chandra RP, Bura R, Mabee WE, Berlin A, Pan X, Saddler JN (2007). Substrate pretreatment: the key to effective enzymatic hydrolysis of lignocellulosics?. Adv Biochem Eng Biotechnol.

[3] Galbe M, Zacchi G (2007). Pretreatment of lignocellulosic materials for efficient bioethanol production. Adv Biochem Eng Biotechnol.

[4] Hu F, Ragauskas A (2012). Pretreatment and lignocellulosic chemistry. Bioenerg Res.

[5] Pienkos PT, Zhang M (2009). Role of pretreatment and conditioning processes on toxicity of lignocellulosic biomass hydrolysates. Cellulose.

[6] Jönsson LJ, Alriksson B, Nilvebrant NO (2013). Bioconversion of lignocellulose: inhibitors and detoxification. Biotechnol Biofuels.

[7] Nilvebrant N-O, Persson P, Reimann A, de Sousa F, Gorton L, Jönsson LJ (2003). Limits for alkaline detoxification of dilute-acid lignocellulose hydrolysates. Appl Biochem Biotechnol.

[8] Cavka A, Jönsson LJ (2013). Detoxification of lignocellulosic hydrolysates using sodium borohydride. Bioresour Technol.

[9] Jönsson LJ, Palmqvist E, Nilvebrant N-O, Hahn-Hägerdal B (1998). Detoxification of wood hydrolysates with laccase and peroxidase from the white-rot fungus *Trametes versicolor*. Appl Microbiol Biotechnol.

[10] Larsson S, Reimann A, Nilvebrant N-O, Jönsson LJ (1999). Comparison of different methods for the detoxification of lignocellulose hydrolysates of spruce. Appl Biochem Biotechnol.

[11] Ximenes E, Kim Y, Mosier N, Dien B, Ladisch M (2010). Inhibition of cellulases by phenols. Enzyme Microb Technol.

[12] Kim Y, Ximenes E, Mosier NS, Ladisch MR (2011). Soluble inhibitors/deactivators of cellulase enzymes from lignocellulosic biomass. Enzyme Microb Technol.

[13] Gellerstedt G, Heitner C, Dimmel DR, Schmidt JA (2010). Chemistry of pulp bleaching. Lignin and lignans—advances in chemistry.

[14] Alriksson B, Sjöde A, Nilvebrant N-O, Jönsson LJ (2006). Optimal conditions for alkaline detoxification of dilute-acid lignocellulose hydrolysates. Appl Biochem Biotechnol.

[15] Alriksson B, Cavka A, Jönsson LJ (2011). Improving the fermentability of enzymatic hydrolysates of lignocellulose through chemical in situ detoxification with reducing agents. Bioresour Technol.

[16] Soudham VP, Brandberg T, Mikkola JP, Larsson C (2014). Detoxification of acid pretreated spruce hydrolysates with ferrous sulfate and hydrogen peroxide improves enzymatic hydrolysis and fermentation. Bioresour Technol.

[17] Sjöström E (1993). Wood chemistry: fundamentals and applications.

[18] Ek M, Gierer J, Jansbo K, Reitberger T (1989). Study on the selectivity of bleaching with oxygen-containing species. Holzforschung.

[19] Bouchard J, Nugent HM, Berry RM (1995). The role of water and hydrogen ion concentration in ozone bleaching of kraft pulp at medium consistency. Tappi J.

[20] Zhang XZ, Ni Y, van Heiningen A (2000). Kinetics of cellulose degradation during ozone bleaching. J Pulp Paper Sci.

[21] Ragnar M. On the importance of radical formation in ozone bleaching. Doctoral dissertation. Stockholm: Department of Pulp and Paper Chemistry and Technology, Royal Institute of Technology (KTH); 2000.

[22] Eriksson T, Ragnar M, Reitberger T (1998). Studies on the radical formation in ozone reactions with lignin and carbohydrate model compounds. Int Pulp Bleach Conf Hels Finl.

[23] Ragnar M, Eriksson T, Reitberger T (1999). Radical formation in ozone reactions with lignin and carbohydrate model compounds. Holzforschung.

[24] Craigee R (1957). The course of ozonization of unsaturated compounds. Rec Chem Prog.

[25] Ragnar M, Eriksson T, Reitberger T, Brandt P (1999). A new mechanism in the ozone reaction with lignin like structures. Holzforschung.

[26] Eriksson T, Gierer J (1985). Studies on the ozonation of structural elements in residual Kraft lignins. J Wood Chem Technol.

[27] Persson P, Andersson J, Gorton L, Larsson S, Nilvebrant N-O, Jönsson LJ (2002). Effect of different forms of alkali treatment on specific fermentation inhibitors and on the fermentability of lignocellulose hydrolysates for production of fuel ethanol. J Agric Food Chem.

[28] Cavka A, Alriksson B, Ahnlund M, Jönsson LJ (2011). Effect of sulfur oxyanions on lignocellulose-derived fermentation inhibitors. Biotechnol Bioeng.

[29] Nilvebrant N-O, Björklund Jansson M. Hydroxyl radical formation during hydrogen peroxide bleaching. Stockholm: STFI-Packforsk Report 197; 2005.

[30] Sluiter A, Hames B, Ruiz R, Scarlata C, Sluiter J, Templeton D (2006). Determination of sugars, byproducts, and degradation products in liquid fraction process samples.

